# Charitably funded hospices and the challenges associated with the COVID-19 pandemic: a mixed-methods study (CovPall)

**DOI:** 10.1186/s12904-022-01070-8

**Published:** 2022-10-10

**Authors:** Ian W Garner, Catherine Walshe, Lesley Dunleavy, Andy Bradshaw, Nancy Preston, Lorna K Fraser, Fliss EM Murtagh, Adejoke O Oluyase, Katherine E Sleeman, Mevhibe Hocaoglu, Sabrina Bajwah, Rachel L Chambers, Matthew Maddocks, Irene J Higginson

**Affiliations:** 1 Division of Health Research, Lancaster University, Lancaster, UK; 2 Health Sciences, University of York, North Yorkshire, UK; 3 Wolfson Palliative Care Research Centre, Hull York Medical School, University of Hull, Hull, UK; 4 Cicely Saunders Institute of Palliative Care, Policy and Rehabilitation, King’s College London, London, UK; 5 King’s College Hospital NHS Foundation Trust, Denmark Hill, UK

**Keywords:** Charitably funded Hospice, COVID-19 Pandemic, Mixed-Methods Research, Funding constraints

## Abstract

**Background:**

Independent charitably funded hospices have been an important element of the UK healthcare response to the COVID-19 pandemic. Hospices usually have different funding streams, procurement processes, and governance arrangements compared to NHS provision, which may affect their experiences during the COVID-19 pandemic. The aim of this study is to understand the challenges faced by charitably funded hospices during the COVID-19 pandemic.

**Methods:**

Eligible Organisations providing specialist palliative or hospice care completed the online CovPall survey (2020) which explored their response to the COVID-19 pandemic. Eligible organisations were then purposively selected to participate in interviews as part of qualitative case studies (2020-21) to understand challenges in more depth. Free-text responses from the survey were analysed using content analysis and were categorised accordingly. These categorisations were used a priori for a reflexive thematic analysis of interview data.

**Results:**

143 UK independent charitably funded hospices completed the online CovPall survey. Five hospices subsequently participated in qualitative case studies (n = 24 staff interviews). Key themes include: vulnerabilities of funding; infection control during patient care; and bereavement support provision. Interviewees discussed the fragility of income due to fundraising events stopping; the difficulties of providing care to COVID-19 and non-COVID-19 patients within relatively small organisations; and challenges with maintaining the quality of bereavement services.

**Conclusion:**

Some unique care and provision challenges during the COVID-19 pandemic were highlighted by charitably funded hospices. Funding core services charitably and independently may affect their ability to respond to pandemics, or scenarios where resources are unexpectedly insufficient.

## What is already known about this topic

Specialist palliative care services pivoted to provide care to both non-COVID-19 and COVID-19 patients as integral parts of a wider healthcare system. Care and advice were provided to patients dying with COVID-19, and those caring for them, including charitably funded hospice services.

## What this study adds

This analysis focuses on the impact of COVID-19 on charitably funded hospices. The emergence of COVID-19 exposed the fragility of charitable hospice funding from the pre-lockdown era and highlighted how the current funding structure may be less suitable during emergency, pandemic conditions.

## How this study might affect research, practice, or policy

This study highlights the challenges faced by Charitably funded hospices, emphasising the unsustainability of their financial model prior to COVID-19, how COVID-19 exacerbated and highlighted this fragility in funding, and how poor funding has increased staff burden and reduced the quality of patient care.

## The Challenges Experiences by Independent Charitably Funded Hospices during the COVID-19 pandemic: A mixed methods study (CovPall)

Palliative care provision has been central to the healthcare response to COVID-19[1, 2]. Globally, palliative care is provided in many ways, with different contexts, organisational and funding arrangements[3]. In many countries, including the UK, there is a mixed economy of provision. Independent, charitably funded hospices have been embedded in mainstream palliative care provision in the UK since the establishment of the first modern hospices[4, 5]. Some care is provided by organisations that are fully publicly funded (e.g. NHS wards or community care teams), and other care provided by independent charitable organisations that typically fundraise to meet around 70% of their costs[6]. It is estimated that 40% of services in the UK are hospital palliative care teams (mainly NHS funded), but 26% are inpatient hospices (mainly charitably funded), and 34% home based teams (variably funded)[7]. Services that are primarily charitably funded are usually independent charitable organisations, part of the local context of health and social care but are run and managed separately. This means they are generally responsible for organising their own staffing, procurement of goods and services, and setting policies and procedures. Palliative care organisations have faced exceptional challenges during COVID-19, it is important to critically analyse if those organisations that are primarily charitably funded have experienced the impact of the pandemic in particular or specific ways.

Data from the multi-national CovPall survey exploring the response of palliative care services to the COVID-19 pandemic indicated that charitably managed services reported less integration with national health services and had a greater likelihood of personal protective equipment (PPE) shortages compared to publicly managed services[2]. Charitably funded hospices in the UK have large numbers of volunteers supporting services[8], and the CovPall survey demonstrated a decline in their deployment during COVID-19[9]. However, there were indications that for charitably managed services busyness increased less than publicly funded services[10]. UK hospices also frequently closed services such as their day hospices early in the pandemic[11]. The purpose of this study is to explore the specific impact of COVID-19 on independent/charitably funded palliative and hospice care organisations in the UK.

## Methods

### Aim

To understand the challenges faced by charitably funded UK hospices in providing palliative and end-of-life care during the COVID-19 pandemic.

### Design

This study adopted a mixed method complementarity approach, with an explanatory sequential design, with the data being merged [12]. A cross-sectional online survey was completed, from which free text responses were collated and used to develop the topic sheet for the case studies with independent charitably funded UK hospices with selected organisations. This is part of the wider CovPall study [1, 2, 9, 10, 14] exploring the multi-national specialist palliative care response to COVID-19.

### Population and setting

Respondents representing specialist palliative and hospice care organisations, providing care in any setting multi-nationally were invited to take part in the survey. For this analysis only those organisations that identified themselves as predominantly charitably funded (received less than 50% of their funding from the NHS) were included.

### Sampling and recruitment

Organisations were invited to take part in the online survey through open advertisement (e.g., via social media) and distribution via palliative care and hospice organisations. Once interest in completing the survey was indicated, information about, and a link to the online survey, was provided to site leads. Potential case study sites (cases defined as English hospices[13]) were identified from survey responses, sampled for maximum variability against the following key criteria: (1) sites in different geographies of the UK; (2) providing a variation in type of and number of services; (3) experiences of caring for COVID-19 patients (discovered in the survey data); and (4) the proportions of minority ethic patients served, until sufficient organisations were recruited[13]. The use of survey data and case studies allowed for triangulation of the data to develop a comprehensive understanding of the challenges faced by charitably funded hospices during the COVID-19 pandemic. Site leads of participating organisations identified potential respondents who could provide rich information about their experiences during COVID-19 from a variety of clinical and organisational perspectives.

### Data collection

Survey data were collected online using the survey website REDCap from 23.4.20 to 31.7.20, and the survey is appended (supplementary material 1) and reported in full elsewhere[2]. Services were specifically asked if their service was managed as a unit that is charitable/non-profit. Demographic data (e.g., number of services provided, number of patient beds on-site, number of COVID-19 cases, region of the UK the hospice is located, if they cared for minority ethnic patients, PPE shortages, and amount of NHS funding relative to total funding) was collected. Within the qualitative case studies semi-structured interviews (conducted between 27.11.20 to 23.3.21) were completed via telephone or online video call on a one-to-one basis, and the topic guide is appended (supplementary material 2). Whilst the topics guided the interview, questions were iterative, and questions directed primarily by participant responses. Interviews were completed by three researchers: IG, AB, and LD, all of whom have interview experience. Moreover, CW is a PhD trained researcher with a nursing background.

All telephone/video calls were recorded and transcribed by a professional organisation verbatim. Notes were made during and after interviews. Interviews lasted, on average, 39 min (ranging from 22 to 80 min).

### Data analysis

Quantitative data were analysed using descriptive statistics. In the online survey participants were asked the free-text response question ‘what do you foresee will be the biggest challenges for COVID-19 in your service over the next 1–2 months’. These responses were reviewed and categorised accordingly using conceptual content analysis. Interview data were analysed using a six step reflexive thematic analysis (RTA) [14]: (1) familiarization of the data; (2) generating codes; (3) deriving themes from the developed codes; (4) reviewing themes; defining and naming the themes; and (6) producing the report. The RTA was completed primarily by the first author (IG) and reviewed by the second author (CW) prior to the analysis being distributed to the research team for final review.

### Ethical issues

Case study sites were only contacted if they reported that they wished to be contacted for this study in the CovPall survey [1, 2, 9, 10, 12]. Eligible organisations were contacted about taking part, and those willing circulated the study details to staff members. Interested staff members contacted the site liaison who forwarded their information to the research team. Prior to taking part, interested participants were given the participant information sheet and consent form and given the opportunity to ask any questions. Prior to the interview commencing participants were given the opportunity to ask any questions and reminded of their rights as participants. Research ethics committee approval was obtained from King’s College London Research Ethics Committee (21/04/2020, Reference; LRS19/20-18541), with additional local approval from Lancaster University (FHMREC 24.11.2020 Reference FHMREC20057). The study was registered on the ISRCTN registry (27/07/2020, ISRCTN16561225).

## Results

A total of 143 organisations identified themselves as managed as a charitable/non-profit unit (rather than publicly or privately funded) in the UK within the online survey. Organisational demographic information is presented in Table 1.

**Table 1:** Demographic Information of Responding Organisations

	n/N (%)
**UK Region**	
England	123/143 (86.0)
Scotland	14/143 (9.8)
Wales	5/143 (3.5)
Northern Ireland	1/143 (0.7)
**Type of Services Provided**	
In-Patient Hospice	127/143 (88.8)
Hospital Pall. Care Advisory	33/143 (23.1)
Specialist Pall. Home Care	97/143 (67.8)
Hands-on Nursing Care	78/143 (54.5)
**Type of Patient(s) cared for**	
Adult only	119/143 (83.2)
Children only	13/143 (9.1)
Adult and Children	9/143 (6.3)
Missing	2 (1.4)
**Number of organisations reporting COVID-19 Cases**	
Staff COVID-19 cases	131/143 (92.3)
Volunteer COVID-19 cases	30/143 (22.1)
Patient COVID-19 cases	127/143 (97.9)

Table 2 provides the demographic information of the five organisations who took part in the case study interviews.

**Table 2:** Contextual Information of the Five Organisations

	N of interviews conducted	Total n of service types provided	Total in-patient Beds	COVID-19 Cases	Minority ethnic patients cared for	PPE Shortages reported	NHS Funding (%)
**O1**	3	4	13	32	Yes	Yes	40
**O2**	3	2	13	0	No	Yes	25
**O3**	6	3	45	12	Yes	Yes	34
**O4**	6	4	18	80	No	Yes	30
**O5**	6	2	---	120	Yes	No	24

Volunteers provided direct patient/family support, indirect patient/family support, back-office functions, and worked as shop volunteers in all sites. Only two hospices cared for both adult and child patients, with the remaining four sites caring for adult patients only. Number of in-patient beds varied between sites, with one organisation not stating the number of beds available (see Table 2).

Of the 143 charitable hospices in the survey who identified as charitable/non-profit units, 130 responded to a free text question asking about the key challenges their organisation may face in the future. Table 3 presents the name and description of each response categorisation and the number of sites who stated this as a key concern. Providing patient care and sourcing income due to significant reductions in income were reported the most frequently within the online survey.

**Table 3:** Key Issues facing Charitable Hospices

Category	Description	n/N (%)
Patient Care	Caring for non-COVID-19 and COVID-19 patients in a small facility; resuming closed services; supporting isolated patients.	63/130 (47.7)
Sourcing Income	Considering avenues to increase funding as most funding methods (e.g., on-site retail stores and fundraising events) ceased.	47/130 (36.2)
Staff Workload	Significant increase in referrals and workload placed on staff.	26/130 (20.0)
Staff Shortage	Staff and volunteers shielding, furlough and redundancies, and increasing demands on staff creating large staff shortages.	25/130 (19.2)
Sourcing Equipment	PPE and technology shortages to allow staff to work. Charitable hospices not part of NHS procurement at time of survey.	19/130 (14.7)
Bereavement Burden	Expecting significant increase in use of bereavement services	12/130 (9.2)
COVID-19 Infections	On-site and in the local community, and how this will affect services in the future.	11/130 (8.5)
Patient Visitation	Considering how to allow for patient visitation to resume despite the presence of COVID-19.	9/130 (6.9)

Many of these areas were evident within the analysis of qualitative interview data, with three important themes of vulnerabilities of funding, the challenges of infection control and bereavement burden.

### Vulnerabilities of funding

Organisations reported around 24–40% of their funding came from the NHS; the majority of funding was independently raised, often through fundraising activities and initiatives such as charity retail stores. However, charity shops were required to close and fundraising activities stopped due to pandemic restrictions, so the main existing sources of income for organisations were not available:

*“We fund our care mostly by ourselves, and our charity has a lot of shops as well, and a lot of money is coming from these shops, and we had to close them and we had … a lot of money was lost from the shops, so we had to furlough a lot of people, as well”* – O5, P1

*“Yeah, well, our funding comes from three streams; a third comes from the NHS, a third comes from things like legacies and other fundraising events and another third comes from the shops but obviously with the shops, and we’ve probably got about 12 shops, with them being shut a lot of the year then that revenue dried up.”* – O2, P33

One site noted how implementing necessary changes and a loss of patients meant they were operating at a £2 million deficit, and had to initially furlough staff, and eventually make 10% of staff redundant:

*“We had a financial major challenge on our hands, we were looking at a £2 Million operating loss so we decided to maximise the number of staff that we could put on furlough, but by nature that meant less creative heads to think about how to respond for those families.”* …….*“Now saying that we’ve brought the staff back in now although we’ve also had to make £600,000 worth of staff redundancies across the organisation, which is about 10% of the staffing complement”* – O4, P16.

*“Yeah, I think the thing that probably we haven’t brought out is some of the knock-on effects of redundancies, furlough, people no longer working with us, because their services are…”* – O3, P8

Whilst other sources of funding did become available during the pandemic, the pandemic related experiences highlighted and exacerbated the fragile funding position of most participating organisations:

*“I mean, we’ve always said that from the beginning but it’s really shown it [the fragility of funding] in a pandemic, you’re having to make people redundant, it’s awful, yeah.”* – O4, P20

These findings emphasise the vulnerabilities of funding for charitably funded hospice, how the COVID-19 pandemic and lockdown(s) exacerbated these issues, and potentially the unsustainability of current models of funding.

### The challenges of infection control

Whilst infection control given a highly transmissible virus is problematic across all settings, participating organisations highlighted particular issues related to typically operating within smaller buildings with often only a single in-patient unit and staff team. Participants spoke about how the hospice altered areas for confirmed COVID-19 and non-COVID-19 patients, and how staff would be separated to work in specific areas (i.e., treating COVID-19 patients or non-COVID-19 patients only):

*“Well, it’s been difficult really because not only have we had to cohort the ward, so finding two separate teams, you know, a hot [COVID-19] side and a cold [non-COVID-19] side, but a lot of the staff have been shielding. A lot of them have been isolating. A lot of them have had COVID”* – O2, P31

*“None of us are allowed onto the ward; they have quite strict guidelines for going on the ward.”* – O5, P10

The relatively small size of the clinical teams working within hospices meant that there were challenges sustaining an appropriate response from infection control perspectives because of their relative inability to provide cross-cover if staffing was affected by COVID-19 infection or isolation:

*“Yeah. I remember I think I was on nights and it was the first time it had happened because, yeah, we might have had two nurses on the night shift or something like that. And sort of 18 patients. And I had the COVID patient. Or I think there was two COVID patients. I had to take like five other patients. And I ended up ringing the senior sister and just saying, “You know, I don’t feel comfortable with this, I don’t feel comfortable, I don’t really want to do it”. And unfortunately, we didn’t have any other – we didn’t have enough staff, so I had to.”* – O3, P2.

It is, therefore, unsurprising that attempting to separate COVID-19 and non-COVID-19 patients, often within the same ward area, was not sufficient to prevent staff and patients contracting COVID-19. Indeed, one participant stated that, on reflection, infections were inevitable:

*“Most hospices in X have had to shut down repeatedly because they’ve had outbreaks in their in-patient units, which I think is inevitable – I mean outbreaks all over the hospitals – but one of the frustrating things for hospices is that whilst we are now under the CQC hospital inspection regime we remain under the community rules for outbreaks for Covid so that’s like a care home”* – O3, P16.

*“You know, and one point near Christmas we had no patients because we had an outbreak. And all the patients had to go. A staffing outbreak”* – O2, P31

These findings highlight the difficulty of attempting to provide care to COVID-19 and non-COVID-19 patients in a relatively small hospice building with limited staff, and how charitable hospices lacked the necessary information and resources to effectively manage these demands. This meant changes to the typically ‘gold standard’ of palliative care that charitable hospices aim to provide were reduced to ‘bronze’ or ‘silver’ standard:

*“We have hairdressers that come in, there isn’t anything and it puts a bit more pressure on the nursing teams as well because, you know, we’re sort of we’re doing our own jobs but we’re doing partial jobs of a lot of other people, you know, even just things like the drinks trolley and stuff was always run by volunteers through the day whereas we take that on now and we’re on minimum staff sometimes to the point where, you know, they’ve been offering us crazy hours to work just to try and cover the shifts, would you like to do an overlap and, yeah, it’s… things are tricky.”* – O4, P21

When discussing the impact of furlough, redundancies, shielding of volunteers, and the impact on staff, interviewees spoke about how working staff had to complete tasks typically completed by furloughed staff.

*“Yes, so people have been furloughed, yes. But nursing staff obviously we are not furloughed, the clinical staff are not furloughed unless they have been deemed as clinically vulnerable, and then they have had to shield.”* – O3, P4

*“The other thing we didn’t do was although we furloughed our retail staff obviously because we had the shops closed, we didn’t furlough very many other staff, so I know a lot of other charities and hospices furloughed fundraising staff, we didn’t furlough anybody, and furloughed the teams that manage volunteers, and we didn’t furlough them either.”* – O1, P41

These findings show how Charitably funded hospices had to furlough, make staff redundant, and place additional burden on working staff. Additionally, findings show the challenges of providing care for both COVID-19 and non-COVID-19 patients, particularly in small hospices with few beds.

### Bereavement Support

Participants spoke about how bereavement services were adjusted to distance-support only (such as via phone call or sending out letters), and in some cases, suspended altogether. Participants also spoke about how the adjustments allowed them to provide some bereavement support, the quality of support provided was lacking in comparison to bereavement support typically offered pre-COVID-19:

*“But I made a point when we had these deaths without family members being around that we sort of gave them an extra phone call just making sure they are okay, that they understand what happens. Because the whole process was different. Before, they would come in and collect the certificate, the certificate of cause of death. And that didn’t happen anymore, so it was all, everything was without context now. So, everything was being sent via email to the registrar, the registrar will then get in contact by phone. So, the whole process changed and therefore we didn’t get the opportunity to see them afterwards. And have a sort of one to one”* – O5, P12

*“I think the other thing that we… we do a lot of children’s bereavement counselling and pre-bereavement counselling; that tends to be quite a lot done in groups so that just collapsed. And actually, children and young people really suffered from a lack of access to counselling and bereavement”* – O4, P16

*“Obviously we’ve got a bereavement counselling service and a children’s service and then we had a day service; so unfortunately, our day service essentially had to shut down because you can’t have everybody in a room, but we did then give virtual support for those cohorts, but it was nowhere near the same as what they were getting in terms of…”* – O4, P16 cont.

The issues associated with a lack of suitable bereavement services are exacerbated with families not able to visit the organisations and see their relatives before their death, which is likely to increase the need and demand for bereavement support when services resume.

*“COVID has got in the way a lot and it has been frustrating because you just feel that the patient and the families aren’t getting the best experience and you know from the research that the family’s loss it’s harder for them to grieve and get over the bereavement having not been able to be here and things like that”* – O2, P33

*“The bereavement side of things, there’s going to be an awful lot of bereaved people who haven’t been able to grieve properly with support, and a lot of people would rely on things like bereavement groups to help them through this period”* – O5, P3

The closing of bereavement services may suggest that they were considered non-essential to the running of the service during the COVID-19 era. However, participant discussions on the subject emphasise how important they are for families of patients, and perhaps that the services were shut due to necessity. These findings also show that upon resumption, the demand for bereavement support is likely to be high and place great strain on the organisations.

## Discussion

Charitable/non-profit hospices and palliative care services within the UK experienced particular challenges during the COVID-19 pandemic with funding vulnerabilities, infection control challenges associated with being small teams in relatively small buildings, and the challenges of bereavement support highlighted.

In the UK, despite having a large and comprehensive national health service,the charitable hospice sector manages and funds a large proportion of specialist palliative care provision. It is currently estimated that statutory funding covers just 37% of the costs of specialist palliative care[15]. As demand for palliative care rises due to an ageing population, the sustainability of this funding model is questionable[15, 16], even though the charitable sector helps to reduce strain on NHS services[17]. The COVID-19 pandemic has further exposed the fragility of this highly-debated funding model. The contribution of the charitable sector to providing essential care is not under question, but the scope, focus and sustainability of these funding models are. There are debates about the ‘exclusivity’ of charitable hospice care providing ‘a little bit of heaven for the few’[5], struggling to reach some populations[18], and the ethical and moral issues of fundamental care being charitably provided within a state funded system[19]. Despite ongoing debates and proposals regarding funding models, there has been no concerted or systematic change to the current patch work of funding, with the potential (or actual) risk of financial collapse being more present than ever [15, 20]. This is increasingly considered to be unviable, and unacceptable as a mode of provision for essential palliative care[21].

Charitably funded independent hospices typically operate within relatively small stand-alone buildings, not often part of a larger health-care estate. In this study the mean bed size for the hospices that provided in-patient care was relatively small, varying from 13 to 45 beds in our case studies. This restricted the flexibility of hospices in terms of how they could manage some infection control challenges, for example providing care in separate areas dependent on COVID-19 status. The scope of ability to do this effectively is different to NHS care provision, where hospitals were rapidly re-configured to cohort those known to have COVID-19 in different areas, provide surge capacity, and with major re-deployments of staff between departments[22]. Despite these perceived challenges, evidence suggests that a smaller setting may be an advantage in terms of controlling the spread of COVID-19. Clearly, nosocomial infection has been a major challenge within the NHS [23]. The nursing and care home sector have faced similar challenges in terms of size of physical space and staffing requirements, and here there is evidence that COVID-19 outbreaks are more likely in larger, not smaller, homes[24]. The size and design of hospices may, indeed, be a benefit. Close attention to interventions such as ventilation and other measures to limit airborne spread within hospices has a major impact on reducing outbreaks[25], and there is a relatively large amount of ‘private’ space such as single rooms with access to outdoor areas that may mitigate virus transmission[26].

Charitable hospices typically offer a range of services from day hospice, to in-patient care, community care and through to bereavement support. This can lead to extended involvement with patients and their families, which whilst felt to improve care quality, may be more exposing in terms of the burden that people feel if such care cannot be provided in the manner expected[13]. The loss of ‘normal’ bereavement care was clearly felt, particularly in the context of increasing need due to the serial losses that have, and will continue to be, experienced as a result of the pandemic[27, 28]. If hospices are to effectively contribute to addressing issues of complex and complicated grief then they will have to adapt rapidly. This may include growing services, sharing expertise, exploring new ways of working, and seeking funding to ensure that these services are adequately and properly provided[29, 30].

## Conclusion

Charitably funded hospices in the UK operate as both part of systems of palliative care in a locality, but also separately to NHS provision. Some aspects of the COVID-19 pandemic were experienced in particular ways because of this positionality, exposing known vulnerabilities related to fragile funding and sustainable service provision. This further emphasises the need for a whole system response to the provision of properly funded palliative care, not only in terms of a coordinated pandemic response, but also in the provision of excellent holistic care to those with palliative care needs. The environment for palliative care funding and provision will continue to be challenging for the foreseeable future. These data can inform debates about how to ensure continuing, high quality palliative care to patients and their families in a way that draws on the strengths of features of charitably funded hospice care in the UK.

### Limitations

Some case study sites had as few as three participants representing their site in interviews. While case studies are in-depth and require fewer numbers, for these sites we could not interview enough participants within different levels of the organisation hierarchy to develop a holistic understanding of the challenges faced by these sites during the COVID-19 pandemic. Additionally, interviews were completed retrospectively and may recall events different compared to if interviews were completed in real-time.

## Data Availability

Data can be requested for up to 10 years and will be considered on a case-by-case basis on receipt of a methodologically sound proposal to achieve aims in line with the original protocol. The study protocol is available on request with IJH as the point of contact. All requests for data access should be addressed to the Chief Investigator via details on the CovPall website (https://www.kcl.ac.uk/cicelysaunders/research/evaluating/ covpall-study, and palliativecare@kcl.ac.uk) and will be reviewed by the Study Steering Group.
