# Correction: Charitably funded hospices and the challenges associated with the COVID-19 pandemic: a mixed-methods study (CovPall)

**DOI:** 10.1186/s12904-022-01084-2

**Published:** 2022-11-02

**Authors:** Ian W Garner, Catherine Walshe, Lesley Dunleavy, Andy Bradshaw, Nancy Preston, Lorna K Fraser, Fliss EM Murtagh, Adejoke O Oluyase, Katherine E Sleeman, Mevhibe Hocaoglu, Sabrina Bajwah, Rachel L Chambers, Matthew Maddocks, Irene J Higginson

**Affiliations:** 1 Division of Health Research, Lancaster University, Lancaster, UK; 2 Health Sciences, University of York, North Yorkshire, UK; 3 Wolfson Palliative Care Research Centre, Hull York Medical School, University of Hull, Hull, UK; 4 Cicely Saunders Institute of Palliative Care, Policy and Rehabilitation, King’s College London, London, UK; 5 King’s College Hospital NHS Foundation Trust, Denmark Hill, UK

**Correction: BMC Palliative Care 21, 176 (2022)**

**https://doi.org/10.1186/s12904-022-01070-8**

In this article [1] the author name Dunleavy was incorrectly written as Dunleavey.

This has been corrected in this correction and the original article has been updated.
